# MRI-only radiotherapy from an economic perspective: Can new techniques in prostate cancer treatment be cost saving?

**DOI:** 10.1016/j.ctro.2022.11.012

**Published:** 2022-11-22

**Authors:** Emilia Persson, Niklas Svanberg, Jonas Scherman, Christian Jamtheim Gustafsson, Adam Fridhammar, Frida Hjalte, Sven Bäck, Per Nilsson, Adalsteinn Gunnlaugsson, Lars E. Olsson

**Affiliations:** a Department of Hematology, Oncology, and Radiation Physics, Skåne University Hospital, Klinikgatan 5, Lund 221 85, Sweden; b Department of Translational Medicine, Medical Radiation Physics, Lund University, Carl Bertil Laurells gata 9, Malmö 205 02, Sweden; c The Swedish Institute for Health Economics, Råbygatan 2, Lund 223 61, Sweden; d Division of Oncology, Department of Clinical Sciences Lund, Skåne University Hospital, Lund University, Klinikgatan 5, Lund 221 85, Sweden

**Keywords:** Cost evaluation, MRI-only, Synthetic CT, Radiotherapy planning, Clinical workflow, Prostate cancer

## Abstract

**Background and Purpose:**

The aim of this study was to analyze a magnetic resonance imaging (MRI)-only radiotherapy workflow from an economic perspective in terms of reduced time, costs and systematic uncertainties.

**Material/Methods:**

A documented Swedish clinical implementation of MRI-only radiotherapy was used as template for cost assessments compared to a combined computed tomography (CT)/MRI workflow. The costs were taken from official regional price lists from 2021. MRI-only specific quality assurance (QA) was assumed necessary in an initial phase. Treatment plans for target volumes with margins of 5–10 mm were created for ten prostate cancer patients prescribed 78 Gy in 39 fractions. The risk of Grade ≥ 2 rectal toxicity or rectal bleeding was calculated using the QUANTEC recommended NTCP model and costs estimated based on subsequent diagnostic examinations.

**Results:**

The exclusion of the CT-examination and faster target delineation were the main contributors to cost reductions. Additional QA procedures limited the initial cost reduction to 14 EUR/patient. Long-term MRI-only reduced the costs by 209 EUR/patient. Reducing margins resulted in Grade ≥ 2 rectal toxicity or rectal bleeding probability of 9.7 % for 7 mm margin and 6.0 % for 5 mm margin. This margin reduction resulted in an additional cost reduction of 46 EUR/patient.

**Conclusion:**

An MRI-only workflow implementation is associated with reduced costs when the workflow tasks are more time efficient and side effects are reduced as a result of margin reduction. The short-term economic benefits are limited due to extra costs of QA procedures. The economic benefits of MRI-only will make impact first when the workflow is well established, and margin reduction has been included.

## Highlights

• MRI-only workflow for prostate cancer radiotherapy can be cost saving.
• The need for quality assurance during an implementation period limits the short-term economic benefit.
• The major cost savings arrives first when the workflow is well established.

## Introduction

Radiotherapy workflows utilizing magnetic resonance imaging (MRI), with its excellent soft tissue contrast, have been proven successful for target and organs at risk delineation for treatment planning of many anatomies including the male pelvis [1], [2]. Computed tomography (CT) however, remains the mainstay imaging modality for treatment planning. CT is used specifically for providing the density maps of tissues required for absorbed dose calculations. As a result, MRI and CT are commonly used in a combined workflow. Since the CT also defines the used frame of reference, the MR images need to be registered to the CT images. This registration process may introduce systematic geometric uncertainties [2], [3], [4], which should be accounted for in the planning target volume (PTV) margin. To address this issue by avoiding image registrations, there has been a strong drive to derive density maps from MR images, often referred to as pseudo CT, substitute CT or synthetic CT (sCT). The sCT images can replace the CT images and enable so called MRI-only workflows [5], [6]. It has been shown that the systematic image registration uncertainty can be reduced by 1–2 mm in an MRI-only workflow [7]. Recently, the clinical image registration uncertainty based on intraprostatic fiducials was investigated with similar results [3]. In addition, it has been proven that contouring in an MRI-only workflow decreases the clinical target volume (CTV) compared to a combined CT and MRI workflow [8].

Methods have been developed for solving tasks that were previously relying on CT images, such as digitally reconstructed radiographs (DRR) and identification of fiducial markers [9], [10], [11], [12]. MRI-only radiotherapy has been proven successful in several clinical studies of radiotherapy of prostate cancer [13], [14], [15], [16].

Removing the inter-modality image registration uncertainty has been one of the main motivations for MRI-only radiotherapy [17]. However, besides a removed registration uncertainty, there are two main additional arguments for excluding the CT and creating an MRI-only workflow: reduced exposure to ionizing radiation and increased time and cost efficacy [18]. The exposure of ionizing radiation will be reduced with the exclusion of CT, but the dose reduction is small, compared to the total treatment dose the patient receives and is therefore of minor interest.

Regarding the time and cost efficacy, a cost evaluation of converting from a combined CT/MRI to an MRI-only workflow for prostate cancer was recently presented [19]. The report considers economic aspects of investment in buildings and scanners as well as economic changes caused by a practically different treatment workflow. In the long run, the cost for MRI-only radiotherapy was found to be slightly lower than a combined workflow. However, many clinics may not rebuild to enable MRI-only radiotherapy, as assumed in Keyriläinen et al., 2021. MRI is today used as an important imaging modality for many diagnoses in radiotherapy, often in combination with CT. For these clinics, where an MR-scanner is already available, a rebuild would not be required.

In a broader perspective, the cost for a radiotherapy treatment should not only consider the tasks that comes prior to or during the treatment itself. Treating pelvic cancers is often associated with side effects such as acute and late gastrointestinal toxicity, genitourinary toxicity, and erectile dysfunction [20]. These conditions may cause additional hospital visits, additional medications, decreased quality of life and overall increased health care costs.

The increased precision in delineation and decreased uncertainty in the set-up results in a smaller CTV can also be an opportunity to decrease the treatment margins [21], [22]. Smaller treatment volumes will result in less radiation dose to adjacent tissues and organs at risk. Accordingly, less side effects can be expected, which would be beneficial for the patients, assuming the local tumour control remains unchanged. Less side effects can also decrease the cost for the health care [23]. Therefore, the effect of reduced treatment volume is worth studying with respect to what impact it may have on health care costs beyond the budget of the radiation oncology clinic.

The aim of this study was to analyze the costs of a combined CT/MRI workflow in comparison to an MRI-only workflow for treating prostate cancer at a Swedish hospital. Further, using a common and widely studied late term side effect of prostate cancer radiotherapy, i.e., Grade ≥ 2 rectal toxicity or rectal bleeding, the potential reduction of health care costs of MRI-only radiotherapy was assessed.

## Material and methods

Following a documented clinical implementation at Skåne University Hospital [14], [24], all tasks and their associated costs were registered for the MRI-only workflow as well as for the combined CT/MRI workflow. In a first step, the resource use, and the costs for the combined CT and MRI workflow for treatment of prostate cancer were determined. In a second step, the resource use and the costs for an MRI-only based workflow were determined using the same procedure. The cost estimations were made based on official regional price lists from 2021 [25], [26], Region Skåne. The costs (per patient) for the different workflow alternatives were calculated both for an ‘implementation period’ and for a ‘post-implementation period’. A learning phase with additional costs, which ceases by time has previously been noted for implementation of new treatment techniques, e.g., intensity modulated radiation therapy [27]. During the implementation period, quality assurance procedures specific for MRI-only radiotherapy were assumed necessary but excluded for post-implementation. Description of the combined CT/MRI workflow and changes required for the MRI-only workflow are described below. Costs are presented in EUR 2021. All individual tasks used from the price lists are presented in supplementary material 1.

### The CT/MRI workflow

The workflow started with a physician visit and implantation of gold fiducial markers, estimated to include one ultrasound examination of pelvis including a puncture biopsy, performed by a physician. An appointment for patient information was scheduled in combination with the CT and MR imaging sessions. This was assumed to equal a nurse visit in terms of the cost at the radiation clinic. The imaging examinations performed were a CT without contrast agent and an MRI without contrast agent of lower pelvis including the prostate. The identification of gold fiducial markers did not require an additional visit for the patient and was not scheduled as a task, however did require time for a nurse. Therefore, this cost was assumed to equal half the cost of a nurse visit. The target delineation by physician was estimated to 45 min per patient and the treatment planning by nurse and/or medical physicists was estimated to 120 min per patient. The time for target delineation and treatment planning procedures were according to the clinical workflows at the time of investigation. The tasks were in the clinic set to 45 and 120 min respectively, regardless of the actual time spend on each patient. To be consistent with the clinical practice and enable a cost estimation, the respective costs presented in the regional price lists for these tasks were used. After treatment planning, but before treatment start, two clinical routine controls of the treatment plan were performed, i.e., one control by a physicist and one control by a nurse, which were included in the cost calculation. Both controls were assumed to be performed according to clinical practice and included individual checks of the treatment plan and the physicist check included a QC-measurement.

### The MRI-only workflow

The MRI-only workflow, like the combined workflow, started with one physician visit and then another visit including the implantation of fiducial gold markers using ultrasound guidance. The information appointment with a nurse was still needed but the CT-examination was excluded. As in the combined workflow the gold fiducial markers had to be identified. Based on the experience from Persson et al., 2020 [14] this was estimated to be more time consuming, due to added complexity in using only MR-images in the identification process. This task was thereby estimated to equal the cost of a full nurse visit. The validation of the identification of the gold fiducial markers was done by a physicist and set to the cost of a physicists check. In this check, the position of the fiducial markers defined in the MR-geometry by a nurse was validated by the physicist. After the implementation period of the MRI-only workflow, the task of identifying and validating the gold fiducials were assumed to require similar staff involvement as for CT, thus estimated to no additional cost.

Based on the experience from our previous studies [8], [14], the target delineation by a physician was estimated to be less time consuming. The time for the target delineation task at the time of the study was 45 min in our clinical workflow. In the regional price lists, the time for target delineation is given with 15 min increments between 30 and 75 min (see supplementary material for details). The time for target delineation in MRI-only was estimated to decrease from 45 to 30 min, corresponding to the smallest available decrease in time according to the price lists. A single modality workflow is less complex since the simultaneous use of two different imaging modalities and image registrations are avoided and the registration between images does not have to be validated by the physician. The treatment planning by nurse and/or medical physicists was estimated to 120 min per patient, which is identical to the combined workflow. After treatment planning, but before treatment starts, the cost for two QA procedures identical to the combined workflow were required. The added task QA of synthetic CT in the MRI-only workflow was assumed to be necessary only during the implementation period but after implementation this could be considered a negligible cost or not needed. The QA of the synthetic CT was assumed to be performed by a physicist and was set to equal the cost of a physicist check. Suggested method for the QA was using Cone Beam CT [28].

### MRI-only toxicity cost estimation

The economic impact of variations in treatment margins and reduced toxicity in MRI-only was assessed in a treatment planning study. MRI-only treatment plans for CTV to PTV margins of 5–10 mm were created and optimized for ten prostate cancer patients prescribed 78 Gy in 39 fractions with volumetric modulated arc therapy (VMAT), corresponding to the clinical indication included in previous study of the implemented MRI-only workflow [14]. In this implementation study, the organs at risk were delineated based on the MR-anatomy only and the CTV was delineated on MR-images with a 1 mm margin and a 7 mm PTV margin. Each treatment plan was created manually by the same treatment planner based on clinical constraints. The treatment plans were optimized to reduce the dose to the rectum as low as possible without violating the clinical constraints for the CTV and PTV.

The risk of Grade ≥ 2 late toxicity or rectal bleeding for each plan and patient was calculated using the QUANTEC-recommended NTCP model [29], from now on referred to as “late rectal bleeding”. The mean risk of late rectal bleeding for the study population was estimated by linear regression. The cost of late rectal bleeding for each PTV margin was calculated by multiplying the estimated mean risk of the side effect for the study population with the costs related to the required hospital visits and diagnostic examinations, visits for prescription of medication and follow up. The examinations included an initial physician visit in the urology department, followed by a proctoscopy, as recommended in the Swedish National care program for prostate cancer [30], and a physician visit to determine presence of the side effect. The cost of a physician visit for treatment or medication prescription and a follow up visit were assumed necessary for the cases where late rectal bleeding was detected. The cost for medical or pharmaceutical treatment of the side effect and change in quality of life were not considered in the calculations.

## Results

**Fig. 1:**
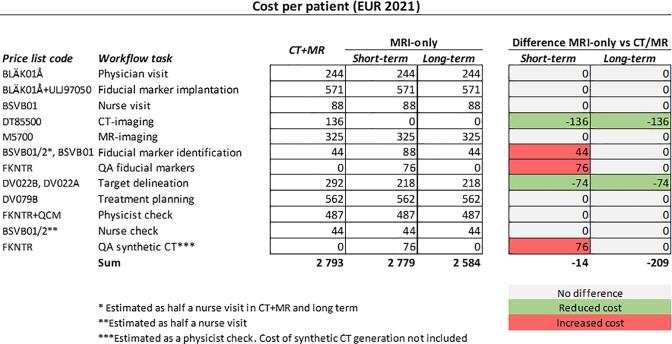
The health care costs (EUR 2021, assuming 1 SEK = 0.1 EUR) for a combined computed tomography (CT)/magnetic resonance imaging (MRI) workflow compared to an MRI-only workflow divided in short-term and long-term perspective. Differences between short-term and long-term compared to the combined CT/MRI workflow are showed to the right in grey (no difference), green (reduced cost) and red (increased cost). (For interpretation of the references to colour in this figure legend, the reader is referred to the web version of this article.)

Associated costs per patient are listed for the combined CT/MRI workflow and the implemented MRI-only workflow (Fig. 1). The excluded CT-examination and the faster target delineation were the main contributors to the cost reductions. Additional QA procedures limits the short-term cost reduction to 14 EUR/patient. On long-term use, assuming a more time efficient workflow in combination with excluded extra QA, costs were reduced by 209 EUR/patient.

**Fig. 2:**
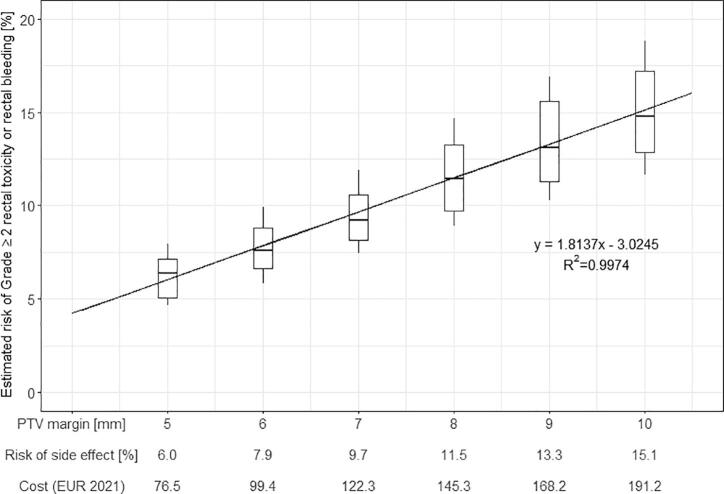
Risk of Grade ≥ 2 rectal toxicity or rectal bleeding (rectal bleeding), calculated using the QUANTEC-recommended NTCP model [29], for PTV margins of 5–10 mm for ten patients. The risk of the side effect for each margin calculated using the given linear regression and the associated costs (EUR 2021, assuming 1 SEK = 0.1 EUR) for required diagnostic examinations and hospital visits are given on the x-axis. Box plots show the distribution for the study population and the medians are presented as black horizontal lines in the boxes. The black diagonal line represents the linear regression with its equation and R^2^ value displayed below the line.

The estimated risk of late rectal bleeding in MRI-only increased for margins increasing from 5 to 10 mm (Fig. 2). Consequently, the health care costs for the diagnosis of late rectal bleeding and follow up hospital visits for potential treatment increased accordingly (Fig. 2). As an example, if a clinic would adopt a margin reduction from 7 to 5 mm due to an MRI-only workflow implementation, the total cost reduction of health care costs would be 46 EUR per patient. The corresponding cost reduction when adopting a margin of 6 mm instead of 7 mm would be 23 EUR. For calculations of other margin scenarios, the reader is referred to Fig. 2.

## Discussion

In this study we have found that MRI-only radiotherapy of prostate cancer can reduce health care costs compared to radiotherapy using a combined CT/MRI workflow. From an economic standpoint the reduction is rather limited initially when costs specific for implementation of MRI-only radiotherapy needs to be added. Once the workflow is optimized to the clinical routine and extra QA procedures are removed, the costs are further reduced. The implementation phase needs to be determined by the local clinic. Therefore, depending on its previous experience, this work will be of different extent and prolongation. However, the economic impact will be substantial first when the workflow is established and the opportunity of the improved geometric accuracy is considered, i.e. reduced treatment margins are applied. Less side effects can be expected from a smaller PTV, with a reduction of health care costs as well as increased quality of life for the patient. However, margin reduction should only be performed after careful considerations of how MRI-only radiotherapy affects the target delineation process and reduction in registration uncertainty. Treatment of a too small volume could lead to unintentional undertreatment and in the long run cancer recurrence and hence increased patient suffering and health care costs.

In a recent study, the economic impact of converting from a combined CT/MRI workflow to an MRI-only workflow was evaluated [19]. This study included aspects of investment in new buildings and MR-scanners as well as a reduction of the need for CT-scanners. Access to MRI for radiotherapy clinics was in a recent survey [31] reported to be high, which show that MRI is an extremely valuable imaging modality for treatment planning even without MRI-only radiotherapy. Therefore, the current study takes a different approach with the purpose to answer the question “What is the motivation to implement MRI-only radiotherapy for a radiotherapy clinic in terms of potential cost reductions?”. With prostate radiotherapy as a starting point, Keyriläinen et al., 2021 [19] found that there was a potential for cost reductions, even if there for some tasks were higher costs initially due to increased amount of QA. Identification of fiducial markers in MR-images can be a demanding task [9] and may be one factor for increased QA. Automated methods, e.g., based on artificial intelligence [10], may be useful for this purpose and can be expected to take bigger part in future radiotherapy workflows.

In our calculations the cost for generation of the sCT was not included. This is in accordance with the method presented by Keyriläinen et al., 2021 [19]. However, it is important to note that the cost of the sCT generation should be added to enable a final cost estimation. At present, there are only three vendors providing methods for sCT generation in the pelvis, i.e., Spectronic Medical AB “MriPlanner”, Sweden, Philips Medical “MR-CAT” (The Netherlands) and Siemens Healthineers “Zyngo”, Germany. In addition, some centers seem to prefer in-house solutions [13], [15]. The use of in-house methods will impact the workflow cost differently to when a commercial solution is purchased. As the cost of the sCT generation can wary widely based on the choice of method the presented calculations did not include the sCT generation costs. The present calculation instead gives the radiotherapy clinic an indication of what a sCT generation method may cost to ensure a cost benefit when the final MRI-only workflow has been implemented. The fiducial marker identification and validation are tasks that on short-term results in additional costs. On longer term, no difference is expected as these tasks are assumed to be clinical routine without additional work required compared to the combined workflow. Image guided radiotherapy of the prostate only is recommended by the ESTRO ACROP guideline to be performed on the prostate itself, where different methods can be used [32]. Soft tissue-based registration towards cone beam CT has been suggested for MRI-only patients [33] where the MR-images are used as positioning references. By removing the need for fiducial markers and associated tasks, this solution would reduce the short-term MRI-only costs as the need for fiducial identification and validation is removed. Other potential differences associated with soft tissue match as a substitute to fiducial markers would have to be evaluated and incorporated in the analysis to investigate the impact on overall workflow cost.

There are several limitations of this study. The data were taken from a single clinic in a specific country. The workflow cost is only assessed for the preparation of the treatment and not the actual treatment. Obviously, fractionation schemes vary between clinics and the total cost of the treatment will be dependent on the number of fractions. Compared to the previous study [19] this study presents a narrower scope, specifically investigating the economic effects of the implementation of an MRI-only workflow.

The major argument to convert to an MRI-only workflow, at least from a health care and patient perspective, is to improve the treatment for the patient. Therefore, it is important to study what impact a change in adverse outcome may have on the health care costs. We used grade > 2 late toxicity or rectal bleeding in our calculations as this is a common side effect in prostate radiotherapy which is well studied from a dose-volume perspective. A single treatment planner performed all planning in this study, which could potentially result in bias. This method was chosen in favor of using plans created by different members of staff. Using the same treatment planner ensured the strategy for planning was consistent throughout the study population. At 7 mm, our treatment planning study gave a 9.7 % risk of late rectal bleeding. This is similar to the 10 % risk for the conventional arm that was found in the HYPO-RT-PC trial for their cumulative 5-year late bowel toxicity grade 2 [34]. Other side effects may also decrease when treatment margins are reduced, such as urinary problems and/or erectile dysfunction, which could result in additional cost reductions. However, these side effects were not included in the current calculations. Further medical or pharmaceutical treatment of the side effect and improvement in quality of life were not considered. Therefore, this study likely underestimates the economic impact of implementing MRI-only.

## Conclusion

The implementation of an MRI-only workflow as presented in this work is associated with reduced costs due to exclusion of the CT-examination and time efficacy compared to a combined CT/MRI workflow, as well as expected reduced side effects due to smaller treatment volumes. For MRI-only radiotherapy to be cost-saving, the cost of the sCT generation should not exceed the total cost reduction. The main contributor of the MRI-only cost reduction is exclusion of the CT-examination and the faster target delineation. On a short-term basis, the economic benefit is limited due to extra costs of QA procedures. The total economic benefits of MRI-only radiotherapy will make impact first when the workflow is well established, and margin reduction has been introduced. The cost benefit of MRI only workflow for prostate cancer radiotherapy will probably be direct transferrable to other radiotherapy applications such as brain, head and neck and rectal cancer.

## Declaration of Competing Interest

The authors declare that they have no known competing financial interests or personal relationships that could have appeared to influence the work reported in this paper.
