# Supplementary material for: MRI-only radiotherapy from an economic perspective: Can new techniques in prostate cancer treatment be cost saving?
Source: Clin Transl Radiat Oncol. 2022 Nov 22;38:183–7. doi: 10.1016/j.ctro.2022.11.012 (PMC9720486; doi:10.1016/j.ctro.2022.11.012)
Supplement: Supplementary data 1 [file mmc1.pdf]

Supplementary material 1

Inputs from regional price lists 2021 (EUR) used for cost assessment of pre-treatment workflow and late rectal bleeding. In columns 1-4, from the left, the following are presented: workflow task, original Swedish task explanation according to the price lists, price list code and price for the task. For comparison, a few examples of different times for the target delineation and the treatment planning tasks are given. In the presented work, the target delineation task was either 30 or 45 minutes and treatment planning was always set to 120 minutes. References to the regional price lists are given below the table.

Inputs

Pre-treatment workflow cost assessment

**Task**	**Original swedish task explaination in price list**	**Price list code**	**Price**
Physician visit	Läkarbesök, återbesök, onkolgi/strålningsfysik	BLÄK01Å	244
Nurse visit	Besök, annan HS-personal	BSVB01	88
Physicist check	Fysikerkontroll per fall	FKNTR	76
Nurse check*	-	BSVB01/2	44
Ultrasound of the pelvis with biopsy	Ultraljud lilla bäcknet med punktion/biopsi	ULJ97050	327
MR-imaging prostate	MRT prostata	M5700	325
CT-imaging lower abdomen	DT nedre buk/bäcken, utan iv konstrast	DT85500	136
QC-measurement	QC-mätning/fall	QCM	411
Target delineation, 30 minutes	Definition av targetområde/fall, 30 minuter	DV022A	218
Target delineation, 45 minutes	Definition av targetområde/fall, 45 minuter	DV022B	292
Target delineation, 60 minutes	Definition av targetområde/fall, 60 minuter	DV022C	366
Target delineation, 75 minutes	Definition av targetområde/fall, 75 minuter	DV022D	440
Treatment planning, 60 minutes	Dosplan typ A, 60 min	DV079A	317
Treatment planning, 120 minutes	Dosplan typ B, 120 min	DV079B	562
Treatment planning, 180 minutes	Dosplan typ C, 180 min	DV079C	808

Late rectal bleeding cost assessment

**Workflow task**	**Original swedish explaination in price list**	**Code in price list**	**Price**
Physician visit (Urology department)	Läkarbesök (Verksamhetsområde urologi)	BLÄK01	233
Proctoskopy	Rektoskopi	UJG02	247
Physician visit, proctoskopy	Läkarbesök, nybesök, kirurgi	BLÄK01N	320
Total cost late rectal bleeding		BLÄK01+UJG02+BLÄK01N+2*BLÄK01	1 265

*Nurse check was estimated as half a nurse visit

References:

Regionala priser och ersättningar för Södra sjukvårdsregionen 2021
https://sodrasjukvardsregionen.se/avtal-priser/regionala-priser-och-ersattningar/

Bild- och funtionsmedicin, prislista 2021, Region Skåne
https://vardgivare.skane.se/patientadministration/avgifter-och-prislistor/prislistor-bild-funktionsmedicin/
